# Special Issue “New Developments in Natural Killer Cells for Immunotherapy”

**DOI:** 10.3390/cells12111496

**Published:** 2023-05-29

**Authors:** Jacques Zimmer, Vladimir Jurišić

**Affiliations:** 1 Department of Infection and Immunity, Luxembourg Institute of Health, House of BioHealth 1, 29 rue Henri Koch, L-4354 Esch-sur-Alzette, Luxembourg; 2 Faculty of Medical Sciences, University of Kragujevac, RS-34000 Kragujevac, Serbia

Since their formal discovery in 1975, natural killer (NK) cells have always been proposed in the literature as a potential treatment for cancer and viral infections. This recommendation stems from one of their fundamental properties, namely, the ability to kill a wide range of tumor cells of various histologic origins and virally infected cells without the need for prior immunization or activation, as is the case for T cells [1,2]. However, it was a very long journey from idea to clinical implementation and, despite major advances, NK cell immunotherapy still cannot be applied routinely. Nevertheless, progress is tangible, and many NK-cell-targeting molecules and NK-cell-based adoptive cellular products are being evaluated in clinical trials and already in regular use.

Grossly, one can distinguish between methods that (a) aim to harness the patient’s own NK cells and rescue them from exhaustion and inefficiency, and (b) the adoptive transfer of in vitro pre-activated and expanded autologous or better allogeneic NK cells that are then (re)-infused into the patient. Regarding the latter approach, there are several considerable advantages of NK cells over T lymphocytes: first, it is possible to use allogeneic NK cells, as they do usually not provoke cytokine release syndromes and neurotoxicity (which is often the case with chimeric antigen receptor (CAR) T cells). Autologous NK cells from cancer patients are frequently severely exhausted and functionally deficient, and cannot always be re-transformed into strong effector cells, which moreover are likely to be considerably down-modulated by their own inhibitory receptors in an autologous setting. If the allogeneic NK cells (primary cells or a NK cell line such as NK92 or KHYG-1 [3]) are themselves transduced with a CAR, they become as cytotoxic and tumor-cell-specific as CAR-T cells. They can be prepared and stored beforehand and, therefore, serve as an off-the-shelf treatment, readily available immediately when the patient needs them [1,2,4]. In contrast, T cells for adoptive transfer need to be autologous and to be expanded for each patient in a time-consuming procedure, the output of which might come too late. Furthermore, although both types of cells obviously need to be prepared in GMP facilities, the overall cost of CAR-T cells is significantly higher than for CAR-NK cells.

However, major advances are now also common in the adoptive T cell field, with, for example, NKT cells, TCR γδ T cells, and mucosa-associated invariant T (MAIT) cells being considered. Even CAR-macrophages are being developed.

Among the directly NK-cell-harnessing therapies, humanized monoclonal antibodies were the first to be introduced, such as rituximab (anti-CD20), elotuzumab (anti-SLAMF7), trastuzumab (anti-HER2/neu) against some B cell malignancies, multiple myeloma, and certain forms of breast cancer, respectively. They are acting, at least in part, through antibody-dependent cellular cytotoxicity (ADCC) mediated by NK cells. More recently, bi- and trispecific killer engagers (BiKE and TriKE) have been successfully developed [5]. They are composed of single chain antibody fragments (scFv) that usually bind to CD16 on the NK cells and one or two tumor antigens, or contain IL-15 instead of one scFv to maintain the survival and proliferation of the NK cells. The crosslinking obtained induces the activation of NK cells via reverse ADCC. Furthermore, the group of Eric Vivier described trispecific NK cell engagers with scFv targeting the activating receptors CD16 and NKp46 on the effector site and a tumor antigen on the target cell site [6].

Despite all this remarkable progress, we should not forget the hurdles to truly efficient and game-changing therapeutic use of NK cells, because they exist particularly for solid tumors. Whereas expansion and storage are now more or less brought under control, the in vivo persistence and the capacity of the effectors’ migration into the tumor bed still need to be improved.

This Special Issue covers various topics in the study of NK cell activity from seven groups about several novel aspects of immunotherapy based on NK cells, split into two reviews and five research articles. It is known that NK cell activation is based on the balance of activating and inhibitory receptors. Based on these considerations, the examination of NK cell receptors, as well as potential ways of unleashing the NK cells at the tumor site, is reviewed in detail by Mendoza-Valderrey et al. [7]. In the second review, Bashiri-Dezfouli et al., from the Multhoff group, focus on CAR-NK cells and the remaining hurdles to make them even more efficient anti-tumor effectors [8].

In melanoma and in solid tumors, there are limitations of targeted cell therapy due to the inability to identify tumor-specific target antigens, insufficient homing and infiltration of immune cells, as well as dysfunction of immune cells in the immunosuppressive tumor microenvironment (TME). Immunotherapy based on sophisticated and well-designed CAR-NK cells can overcome the immunosuppression that exists in the TME, and this is discussed by Grote et al. [9].

A new principle regarding the isolation of functional human NK cells with optimized cytokine cocktails from peripheral blood (PB)-CD34+ cells is a very interesting option for clinical practice, which showed improved expansion of NK cells from feeder cells co-expressing 4-1BB ligand and membrane-anchored IL-15 and IL-21. NK cells differentiated ex vivo from hematopoietic stem cells (HSCs) with these feeders significantly enhancing NK cell expansion and fully restoring the variability previously achieved using cytokines alone, is described by the Wels group [10]. The findings indeed offer hope that mobilized PB-CD34+ cells expanded and differentiated according to this two-step protocol may be a good source of allogeneic NK cells for adaptive cancer immunotherapy. Montagner et al. [11] present a CAR introduced into the NK-92 cell line and targeting a prostate cancer antigen, whereas Zamai et al. [12] performed studies of activating and co-activating receptors signaling differentially in various NK cell subsets.

In this Special Issue, the function of NK cells in patients with COVID-19 infection is also presented by Bergantini et al. [13]. The results showed that adaptive/memory NK cell frequencies were significantly higher in patients who died of COVID-19 than in survivors. The activation of NK cells occurs via the overexpression of CD69 and CD25 but, in addition, PD-1 inhibitory signaling maintains an exhausted phenotype in NK cells. These findings correlate with increased values of IL-6 in severely ill patients.

Together, these articles show the significant role of NK cells in medicine; however, many aspects about NK cells remain open questions for further research and future applications in everyday clinical practice.
